# Systemic overuse of health care in a commercially insured US population, 2010–2015

**DOI:** 10.1186/s12913-019-4079-0

**Published:** 2019-05-02

**Authors:** Allison H. Oakes, Hsien-Yen Chang, Jodi B. Segal

**Affiliations:** 1 Department of Health Policy and Management, Johns Hopkins University Bloomberg School of Public Health, Baltimore, MD USA; 2 Center for Health Services and Outcomes Research of the Bloomberg School of Public Health, Baltimore, MD USA; 3 Department of Medicine, Johns Hopkins University School of Medicine, Baltimore, MD USA

**Keywords:** Health services research, Overuse, Quality measurement

## Abstract

**Background:**

Overuse is a leading contributor to the high cost of health care in the United States. Overuse harms patients and is a definitive waste of resources. The Johns Hopkins Overuse Index (JHOI) is a normalized measure of systemic health care services overuse, generated from claims data, that has been used to describe overuse in Medicare beneficiaries and to understand drivers of overuse. We aimed to adapt the JHOI for application to a commercially insured US population, to examine geographic variation in systemic overuse in this population, and to analyze trends over time to inform whether systemic overuse is an enduring problem.

**Methods:**

We analyzed commercial insurance claims from 18 to 64 year old beneficiaries. We calculated a semiannual JHOI for each of the 375 Metropolitan Statistical Areas and 47 rural regions of the US. We generated maps to examine geographic variation and then analyzed each region’s change in their JHOI quintile from January 2011 to June 2015.

**Results:**

The JHOI varied markedly across the US. Across the country, rural regions tended to have less systemic overuse than their MSA counterparts (*p* < 0.01). Regional systemic overuse is positively correlated from one time period to the next (*p* < 0.001). Between 2011 and 2015, 53.7% (*N* = 226) of regions remained in the same quintile of the JHOI. Eighty of these regions had a persistently high or persistently low JHOI throughout study duration.

**Conclusions:**

The systemic overuse of health care resources is an enduring, regional problem. Areas identified as having a persistently high rate of systemic overuse merit further investigation to understand drivers and potential points of intervention.

**Electronic supplementary material:**

The online version of this article (10.1186/s12913-019-4079-0) contains supplementary material, which is available to authorized users.

## Background

Overuse—the provision of care where the potential for harm exceeds the potential for benefit—has been cited as a leading contributor to the high cost of the US health care system [1–4]. Overuse is often physically and psychologically harmful to patients [5–7] and is a definitive misuse of resources. Such wasteful utilization helps to explain why health care spending is inconsistently associated with measures of health care quality [8–10]. More is not always better. The identification and elimination of overused services could improve health outcomes and reduce spending, while redirecting important resources toward the delivery of high-value care [11, 12].

The direct measurement of overuse requires researchers to isolate episodes of care in which patients had a test or treatment that was contrary to published recommendations [13]. Recent studies have estimated that 14 to 25% of Medicare beneficiaries and 8% of commercially insured adults experience one or more overuse events per year [14–17]. Related work has found that the volume of low-value services delivered to Medicare patients varies across regions and physician organizations [15, 16, 18–20]. Analyses of individual procedures have discovered heterogeneous trends in overuse over time. Some interventions, such as renal artery stenting, have declined across years, while others have increased or remained stable [21, 22]. Thus, the measurement of individual overused services does little to inform our understanding of overuse as a broad phenomenon.

We previously defined *systemic* overuse as the general tendency of an organization or region to overuse medical services [23, 24]. To measure this concept, we used claims from Medicare beneficiaries and a portfolio of 20 “bellwethers” to create an index that differentiated regions by their propensity to overuse health care services. In this work, we adapt and validate the Johns Hopkins Overuse Index (JHOI) in a commercially insured US population. We then use the JHOI to examine regional variation in systemic overuse and analyze trends over time to inform whether systemic overuse is an enduring problem [25–27].

## Methods

### Data sources and sample population

We used the IBM MarketScan® Research Databases. The Commercial Claims and Encounters Database includes inpatient, outpatient, and prescription drug claims that are submitted by private health plans and employers. These are claims from more than 47 million US residents aged 0 to 64 years old with employer-sponsored private health insurance from all 50 states and the District of Columbia. The databases capture medical claims and spending for employees and their insured dependents.

We analyzed inpatient and outpatient claims in semiannual, 6-month intervals. Beneficiaries were included if they were 18 to 64 years old and had continuous health insurance coverage during the specified time period. For our analysis, individuals were assigned to a Metropolitan Statistical Area (MSA) (*N* = 375) or to the rural region of their state (*N* = 47) based on the geographic location of the primary beneficiary’s residence. Data from 2010 was used to adapt and validate the index, while the data from 2011 to June 2015 was used to examine trends over time.

### Generating the index in commercial claims

#### Adapting the indicator procedures

The JHOI was originally developed and validated in the Medicare population. It includes 20 clinically diverse claims-based measures of overuse that are aggregated into an index [23] (Additional file 1: Table S1). We used three criteria to consistently and transparently assess whether the indicators from the original specification of the JHOI were suitable for use with these new data [28]. We assessed whether each of the indicators was “specifiable” – the indicator can be operationalized in these data; “indicated” – relevant to a 18–64 year old population of beneficiaries; and “variable” – having as much frequency and variance across regions as was observed in the Medicare data [24]. These criteria ensure that each indicator adds reliable and valuable information to the index.

#### Creating the index

We constructed the index as previously specified [16]. First, we created a dataset for each indicator and removed beneficiaries who were ineligible for the indicator procedure (e.g., hysterectomy in men) or in whom use of the procedure would not be considered to be an overuse event (e.g., low back imaging in a patient with metastatic cancer). We then concatenated these data into a single dataset to construct an unbalanced panel. Here we created the dependent variable, denoted *P*_*ijk*_*,* which is equal to 1 if there was overuse in the *i*th beneficiary, as measured by the *j*th procedure in the *k*th region, and equal to 0 if otherwise. Our multilevel model is described in the following equation:

1 $$ {P}_{ijk}={\beta X}_i+{\uppsi}_j+{\Phi}_k+{\upvarepsilon}_{ijk} $$

where *X*_*i*_ is a vector of patient specific factors, ψ_j_ is a set of procedural fixed effects, and Φ_k_ is a set of regional fixed effects. The patient specific factors included age, sex, and a claims-based count of diseases derived from the Agency for Healthcare Research and Quality (AHRQ) Clinical Classifications Software (CCS) for single-level diagnoses [29]. From each model, the Φ_k_ estimate represents the systemic tendency for the *k*th region to overuse across the *j* procedures. This measure was then standardized to create the JHOI as a Z-score, where the value of the index for the *k*th region is estimated as:

2 $$ {\mathrm{JHOI}}_k=\left({\Phi}_k-\overline{\Phi}\right)/\mathrm{SD}\left(\Phi \right) $$

where $\overline{\Phi}$ is the average of the 422 regional fixed effects and SD(Φ) is the standard deviation across the fixed effects that were estimated in Eq. 1. Through standardizing the index, we also created a test statistic. We considered regions with Z-scores more extreme than 2.0 or − 2.0 to be outlier regions.

To examine the effect of risk-adjustment on variation in the index, we added each of the patient specific factors to Eq. 1 in a step-wise fashion. First, a model without risk-adjustment was used, then models with patient demographics and clinical co-morbidities were created. To make a final determination on risk-adjustment, we examined changes in each model’s R-squared and calculated the Spearman rank correlation between the different versions of the JHOI_*k*_.

#### Validating the index

Given the definition of overuse, we hypothesized that systemic overuse should be associated with higher aggregate costs and without clinical benefit in a region. With Spearman rank correlation, we assessed the relationship between regional systemic overuse (JHOI_*k*_) and regional measures of total costs and total mortality. Because Medicare spending is less susceptible to price and local market effects, costs were tabulated using publicly available Medicare files [30]. Total regional mortality was calculated for the 18–64 year old population using publicly available information from the Centers for Disease Control and Prevention’s Wide-ranging ONline Data for Epidemiologic Research (CDC WONDER) database [31].

### Geographic variation and trends

The JHOI_*k*_ were generated for each 6-month period, from January 2011 to June 2015. To illustrate geographic variation, we generated a map displaying the JHOI_*k*_ for each half year period with each region assigned to a quintile based on its Z-score.

To examine changes across the 4.5 year period, we calculated the Spearman rank correlation for each JHOI_*k*_ compared to the base period, which was the first half of 2011. Additionally, we generated a “difference score” for each region by subtracting the JHOI_*k*_ quintile for each time period from the prior time period. These values were then summed in order to estimate *net* change within a given geography across all years of observation. To visualize geographic patterns in net change over time, we generated a map of the difference in the JHOI_*k*_ quintile between the first and last time period. We then computed the proportion of regions that had persistently high or persistently low systemic overuse, defined as having a JHOI_*k*_ in the top or bottom quintile for *all* time periods. We utilized a lasagna plot to visualize region-specific trajectories across study duration [32].

Analyses were performed using SAS version 9.4 and Stata 14 software. Maps were created using the spmap command in Stata [33, 34]. Data from South Carolina are not displayed in maps due to contractual restrictions. Our study was determined to be exempt from review by the Johns Hopkins Bloomberg School of Public Health Institutional Review Board (IRB8729).

## Results

The final sample included more than 300 million beneficiary-level observations from 2010 to June 2015, with an average of 27,366,474 individuals per time period. The data included information for beneficiaries in all possible geographic units, with an average sample size of 69,331 beneficiaries and a minimum of 1087 beneficiaries per region. The total sample was 52% female and had an average age of 42.3 years (SD 13.6). Across the study period, approximately 86% of beneficiaries resided in an MSA. As required, age ranged from 18 to 64 years old and beneficiaries had a full 6 months of insurance coverage during a specified time period.

### The JHOI in commercial claims

Among the 20 indicators that were used to measure systemic overuse with Medicare claims [16, 24], we found that 19 of these 20 procedures met our criteria for application to a younger, commercially insured population. The indicator entitled “More than 1 emergency department visit in the last 30 days of life” could not be operationalized in these commercial claims due to a lack of information on deaths outside of the hospital. The remainder of the 19 procedures could be specified in this new data, are clinically relevant to the 18–64 year old population, and had satisfactory frequency and variation across regions (Table 1). The retained 19 indicators included 13 diagnostic tests, 2 tests used for screening, 1 for monitoring, and 3 therapeutic procedures. The clinical areas included 4 relevant to primary care practice, 3 to otolaryngology, 3 to radiology, 2 to cardiology, and 1 each to neurology, emergency practice, allergy, oncology, urology, physical therapy, and surgery. During each semiannual period, an average of 4.0% (range: 3.9 to 4.2%) of commercially insured adults experienced one or more of the indexed overuse events. This rate consistently varied by more than fourfold across the country (e.g., 1.7 to 8.2% in January through June 2010). Individuals were subjected to between 0 and 5 of the 19 procedures.

**Table 1:** Mean and Median Counts of Indicators of Overuse Across Regions, 2010 months 1–6 (*N* = 422 regions)

Overuse Indicator	Per 1000				
	Mean	Median	Interquartile Range		
Stress echocardiography in symptomatic or ischemic equivalent acute chest pain^a^	52.4	36.6	0.0	–	72.8
Laminectomy or spinal fusion	0.6	0.6	0.4	–	0.8
Hysterectomy for benign disease	3.5	3.3	2.5	–	4.3
Fiberoptic laryngoscopy for sinusitis diagnosis^a^	2.4	1.6	0.7	–	3.0
Nasal endoscopy for sinusitis diagnosis^a^	21.3	16.4	9.5	–	28.5
Routine monitoring of digoxin in patients with congestive heart failure	18.7	16.6	9.6	–	23.5
EEG monitoring in individuals presenting with syncope^a^	59.6	55.3	39.2	–	75.4
Serological tests for *Helicobacter pylori*	4.2	4.1	2.8	–	5.4
MRI in adults with mild traumatic brain injury^a^	22.9	20.8	7.5	–	31.3
PET, CT, and radionuclide bone scan in individuals with low-risk prostate cancer	112	105	80.3	–	135
Traction for low back pain	222	216	169	–	271
Screening for asymptomatic carotid artery stenosis in the general adult population	1.3	1.1	0.8	–	1.6
Preoperative chest radiography in the absence of a clinical suspicion^a^	135	118	89.6	–	165
Tumor marker studies in asymptomatic women with previously treated breast cancer	289	272	165	–	386
Diagnostic tests, such as immunoglobulin testing, in the evaluation of allergy^a^	12.2	10.5	5.9	–	16.7
Sinus CT for uncomplicated acute rhinosinusitis^a^	1.1	0.9	9.0	–	1.4
MRI lumbar spine for low back pain^a^	491	493	454	–	530
Thorax CT use with and without contrast^a^	70.2	47.4	22.9	–	94.0
Abdomen CT use with and without contrast^a^	182	136	90.6	–	259

*CT* Indicates computed tomography, *EEG* Electroencephalography, *MRI* Magnetic resonance imaging, *PET* Positron emission tomography

^a^Count is per 1000 eligible episodes rather than per 1000 beneficiaries

The index did not significantly change with alternative risk-adjustment methods. The Spearman rank correlation between the unadjusted and the fully risk-adjusted JHOI was high (ρ = 0.98, *p* < 0.0001) (Additional file 1: Figure S1). The regional fixed effects alone accounted for an R^2^ of 18.93%. The addition of patient demographics and clinical co-morbidities only increased the R^2^ by 0.02 and 0.13%, respectively. We opted to use the age and sex-adjusted JHOI for the remainder of our analyses.

In the first half of 2010, the JHOI_*k*_ had a mean of 0 and standard deviation of 1, with a minimum of − 2.60 and maximum of 3.23 (Additional file 1: Figure S2). Regions with a JHOI that was in the upper or lower tail of the standard normal distribution are listed in Table 2. As hypothesized for the validation, the index was positively correlated with total per capita spending on Medicare beneficiaries (ρ = 0.32, *p* < 0.001); annual per capita costs were $1181 higher in regions with a JHOI in the 90th percentile as compared to regions with a JHOI in the 10th percentile. Similarly consistent with our validation hypothesis, the index was not directly associated with an indicator of clinical benefit, operationalized as mortality in the 18–64 year old population (ρ = 0.02, *p* = 0.61) (Additional file 1: Figures S3 and S4).

**Table 2:** Regions with Extreme JHOI Scores, 2010 months 1-6

Region	State	JHOI
Pittsburgh	PA	3.23
Bakersfield	CA	3.11
Oxnard-Thousand Oaks-Ventura	CA	2.68
Lafayette	LA	2.44
Corpus Christi	TX	2.39
Anaheim-Santa Ana-Irvine	CA	2.33
W Palm Beach-Boca Raton-Delray Beach	FL	2.26
Port St. Lucie	FL	2.25
Gulfport-Biloxi-Pascagoula	MS	2.18
Scranton-Wilkes-Barre-Hazleton	PA	2.15
Monroe	LA	2.11
Cape Girardeau	MO-IL	2.02
Sebastian-Vero Beach	FL	2.01
New York-Jersey City-White Plains	NY-NJ	2.01
Bismarck	ND	−2.60
Dothan	AL	−2.46
Rural Region of State	ND	−2.36
Mankato-North Mankato	MN	−2.20
La Crosse-Onalaska	WI-MN	−2.08
Waterloo-Cedar Falls	IA	−2.01

### Geographic variation and trends

**Fig. 1:**
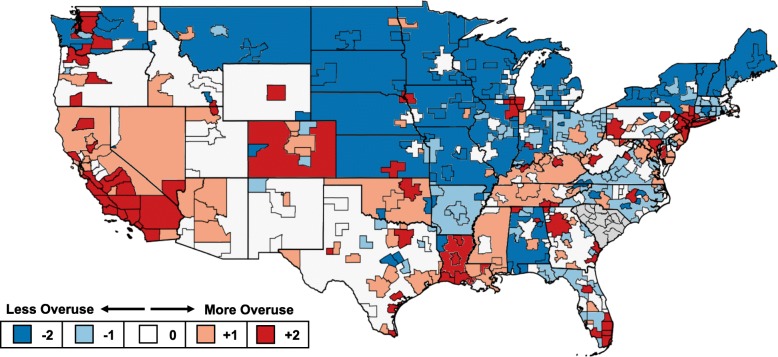
Overall variation in the Johns Hopkins Overuse Index in 2015. Quintiles of the JHOI calculated for each of the 422 MSA/rural region distinctions using commercial claims from the first half of 2015. Data not shown for South Carolina

The JHOI showed marked regional variation (Fig. 1). As illustrated by the data from 2015, systemic overuse was prevalent in a number of coastal metropolitan regions. Examples include multiple MSAs in Washington, California, Texas, Florida, New Jersey, and New York. Six of the top 10 overusing regions were metropolitan areas in California. This group includes the most overusing region, the Oxnard–Thousand Oaks–Ventura, California MSA. While much of the central and northwestern parts of the US had relatively low rates of systemic overuse, Chicago, IL and Pittsburgh, PA are metropolitan outliers with high systemic overuse. Across the entire country, the rural regions of states tended to have less systemic overuse than their MSA counterparts (*p* < 0.01). Illustrating this, only 4 rural regions were in the top quintile of systemic overuse, while 18 rural regions were in the bottom quintile.

**Fig. 2:**
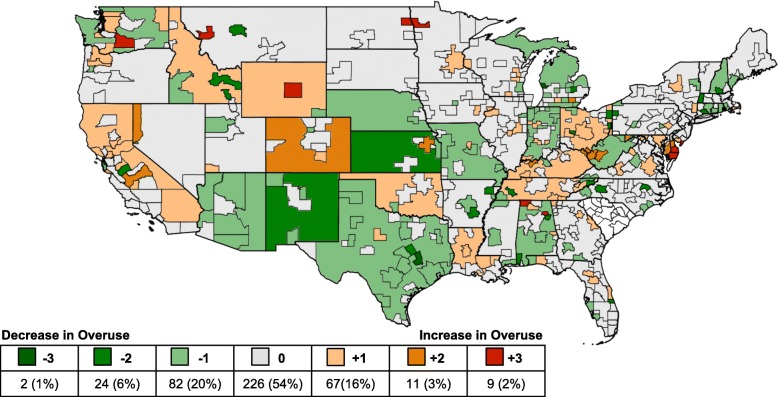
Net change in the Johns Hopkins Overuse Index between 2011 and 2015. This map displays the change in the JHOI quintile from 2011 to 2015 for each of the 422 MSA/rural region distinctions. Data not shown for South Carolina

There was a positive, significant correlation in the JHOI across years. This ranged from ρ = 0.96 (*p* < 0.001) between the first and second half of 2011 and ρ = 0.79 (p < 0.001) between the first halves of 2011 and 2015. The strength of the correlation decreased with the distance between time periods (Additional file 1: Table S2). Across the entire observation period, the average difference score was 0.05 quintiles and the majority of regions, 53.68% (*N* = 226), experienced zero net change. The largest net changes were + 3 quintiles (N = 2 regions) and − 3 quintiles (*N* = 9 regions). A map of the net change in the JHOI between 2011 and 2015 is presented in Fig. 2. Patterns of increasing or decreasing overuse did not differ across rural and urban regions.

**Fig. 3:**
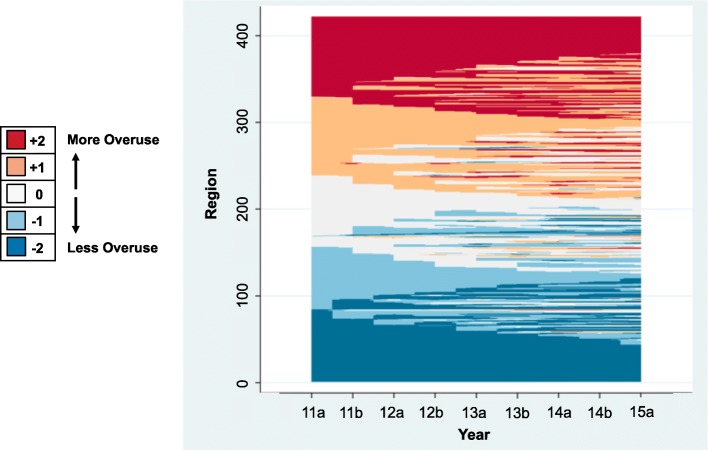
Region-specific changes in the Johns Hopkins Overuse Index from 2011 to 2015. Each region’s trajectory over time is a horizontal layer. The simultaneous plotting of trajectories results in a stacking of layers. Shading is used to represent the 5 JHOI quintiles. The JHOI quintile from the first half of 2011 fixes the vertical position of each region

Numerous regions fell into the top or bottom quintile of the JHOI for *all* time periods (Fig. 3). Specifically, 37 (8.76%) regions had persistently high systemic overuse and 43 (10.18%) regions had persistently low systemic overuse. Only metropolitan areas met criteria for the persistently high overuse phenotype, while 9 rural regions had persistently low systemic overuse. Examples of persistently high overusing metropolitan areas include Pittsburgh, PA, Corpus Christi, TX, Miami, FL, Los Angeles–Long Beach–Glendale, CA, and New York–Wayne–White Plains, NY-NJ (Additional file 1: Table S3).

## Discussion

In the United States, more than 60% of insured individuals have commercial insurance [35]. Despite this, our knowledge about health care utilization is often based on analyses of data from Medicare beneficiaries [36]. In this work, we generated and validated an index to measure systemic overuse in 18 to 64 year old adults with commercial insurance. The JHOI exposed significant variation and revealed a distinct geographic signature of systemic overuse, with higher scores in coastal metropolitan areas and lower scores in central and rural regions of the country. These findings are similar to what has been observed in the Medicare population [15, 16].

Relatively few studies have examined patterns of overuse over time. Those that do tend to focus on a handful of individual low-value services at a national level [21, 22]. One such study analyzed nationwide trends for 16 different services, from 2009 to 2014. Representative of this small but growing area of research, some of the low-value services were successfully deadopted, while many others remained stable or became increasingly common over time [21]. As older, low-value technologies become unpopular there is the potential for new, equally low-value technologies to emerge [37]. While measuring any one specific service may be a valid way to evaluate the impact of a local quality improvement initiative, a single measure cannot reliably characterize overuse as a broad phenomenon. Additionally, any one measure has the potential to be distorted by differences in coding, whether intentional or unintentional.

With a collection of overuse bellwethers, we found that systemic overuse is a resolute problem. From 2011 to 2015, the majority of regions experienced *zero* change in JHOI quintile, making “consistency” the most common phenotype. This was particularly true in the tails—19% of regions were in the top or bottom quintile throughout study duration. The 37 regions that had persistently high rates of systemic overuse support the concept of a distinguishable “culture of overuse” [2, 13, 16]. These findings should encourage policy makers and health system leaders to address structural and system-wide drivers of overuse, which may be more impactful than a focus on individual overused procedures.

By our adapting an index that was originally developed in the Medicare population, there is now a validated tool to measure systemic overuse across the adult lifespan. This work adds to the growing literature on the direct measurement of overuse in claims data. While an indirect assessment of waste that is based on variation in risk-adjusted utilization or spending can provide information on the potential scale of the problem, these methods cannot distinguish between variation in underuse or overuse [13, 17]. For this reason, direct measures are better able to differentiate between more and less efficient regions. Moreover, we found that the measurement of systemic overuse is not meaningfully impacted by differences in patient health status. Taken together, the observed variation in the JHOI indicates that there is substantial room to reduce overuse.

This paper is not without limitations. While some may not agree with each of the included indicators, they were selected because they were identified by reputable professional bodies as being overused services (Additional file 1: Table S1). Additionally, this set of indicators includes diverse clinical activities and each indicator itself is regionally variable. We recognize that some fraction of events may have been misclassified as overuse, but it is unlikely that these clinical encounters would occur in a way that would systematically bias the JHOI or alter its interpretation, particularly given that we are reporting relative differences between regions. As the literature on overused procedures continues to grow, the included indicators might even be swapped out, similar to how the Consumer Price Index regularly changes what is in its basket of goods.

The studied population is comprised of enrollees from large employers and group health plans. While the data include close to 25% of the 18 to 64 year old population with employer-based health insurance in the US [38], our analyses may not be generalizable to the entire commercially-insured US population. We are also unable to control for differences in patient preferences regarding receipt of health care, however prior research has indicated that this is unlikely to be a strong driver of variation in overuse [39, 40]. While not perfectly representative, the included population raises intriguing operational questions; we would expect that self-insured employers have the motivation and power to reduce health care inefficiencies among their beneficiaries. A minor limitation is that we had to allocate beneficiaries to regions based on their primary residence rather than on where the indexed care was received. To the extent that residents of rural regions may travel to urban areas to receive care, our analyses would underestimate the association between metropolitan areas and systemic overuse.

Finally, the JHOI is a *relative* measure of systemic overuse—it compares the extent of health care overuse in one area relative to the national average. This precludes us from being able to make statements about the absolute magnitude of systemic overuse. It also prevents a direct analysis of the costs that are associated with this phenomenon. While we have demonstrated that the JHOI is associated with increased health care spending, it is infeasible to directly measure the entire universe of overused procedures and their downstream consequences. Further, overuse of healthcare contributes to patient harm in a way that may not be directly quantifiable.

Several extensions of this research would be useful. While we demonstrated that systemic overuse varies regionally, it may be informative to examine patterns of systemic overuse across different insurance types, different systems of care, and different patient populations. Payer type and insurance status do not seem to influence rates of overuse of individual services, and we would expect these trends to hold when overuse is more broadly measured [41–43]. We identified regions that had persistently high or persistently low systemic overuse; investigating the drivers of this phenomenon is essential. Studies in the Medicare population have identified associations between overuse and certain regional characteristics of the health care system, such as the number of specialists in an area, the supply of hospital resources, and physician concentration [15, 19]. It would also be worthwhile to examine the few regions that experienced a meaningful reduction in systemic overuse. As measures of overuse become an increasingly common component of performance measurement, additional research is also needed to understand the relationship between the JHOI and other indicators of health care quality [44–46].

This research relies on the premise that geography is a useful unit of analysis for policy. While this work highlights meaningful variation in wasteful health care utilization at the level of the MSA, it does not necessarily mean that this unit of analysis is the right unit for an intervention related to systemic overuse. Aggregating at any level of geography is likely to minimize the true variation that exists and pull the extremes to the regional mean—clinicians within any geographic area are known to have different practice patterns [18, 20, 36, 47, 48]. There are many reasons to believe that health care systems or networks of clinicians might be better targets for intervention. With that said, choosing to implement a national, state, or system-level policy involves tradeoffs related to administrative burden and need for program specificity [49]. Operationalizing the JHOI across and within multiple geographic and non-geographic units of analysis may inform the deployment of interventions to reduce systemic overuse.

## Conclusions

We demonstrated that the measurement of systemic overuse is feasible in commercial claims data. We found that systemic overuse varies across geographies, and that a number of regions have a distinguishable culture of overuse. These findings are similar to those of previous studies among older adults and children, which suggests that interventions to improve care efficiency should include patients across the entire age spectrum [50]. Systemic overuse is a persistent, regional issue which will require experimentation and collaboration by many different stakeholders.

## Additional file

Additional file 1: Description of indicator procedures included in the Johns Hopkins Overuse Index and results from supplementary analyses. (PDF 249 kb)

## Abbreviations

AHRQ: Agency for Healthcare Research and Quality
CA: California
CCS: Clinical Classifications Software
CDC: Centers for Disease Control and Prevention
FL: Florida
JHOI: Johns Hopkins Overuse Index
MSA: Metropolitan Statistical Area
NJ: New Jersey
NY: New York
PA: Pennsylvania
SD: Standard deviation
TX: Texas
US: United States
WONDER: Wide-ranging ONline Data for Epidemiologic Research
