# Supplementary material for: Systemic overuse of health care in a commercially insured US population, 2010–2015
Source: BMC Health Serv Res. 2019 May 2;19:280. doi: 10.1186/s12913-019-4079-0 (PMC6498548; doi:10.1186/s12913-019-4079-0)
Supplement: Supplementary file 1 — Description of indicator procedures included in the Johns Hopkins Overuse Index and results from supplementary analyses. (PDF 249 kb) [file 12913_2019_4079_MOESM1_ESM.pdf]

Online Supplementary File

Systemic Overuse of Health Care in a Commercially Insured US Population, 2010 to 2015

Table 1. Procedures included in the Johns Hopkins Overuse Index

Indicator	Procedure	Reference
Stress echocardiography for detection of CAD/risk assessment in symptomatic or ischemic equivalent acute chest pain (i.e. Acute Coronary Syndrome)	Individuals with CPT codes as listed or HCPCS codes as listed for echocardiography	American College of Cardiology Foundation, American Society of Echocardiography, American Heart Association, American Society of Nuclear Cardiology, Heart Failure Society of America, Heart Rhythm Society, Society for Cardiovascular Angiography and Interventions, Society of Critical Care Medicine, Society of Cardiovascular Computed Tomography, Society for Cardiovascular Magnetic Resonance American College of Chest Physicians[1]
Laminectomy and/or spinal fusion	Laminectomy or spinal fusion	National Guidelines Clearinghouse[2]
Hysterectomy for benign disease	Any hysterectomy (not specified for malignancy treatment)	National Guidelines Clearinghouse[2]
Fiberoptic laryngoscopy for patients with a diagnosis of sinusitis	Laryngoscopy with ICD-9 code indicating sinusitis on the same claim	The Alternative Quality Contract[3]
Nasal endoscopy for sinusitis diagnosis	Nasal endoscopy with ICD-9 code indicating sinusitis on the same claim	The Alternative Quality Contract[3]
More than one emergency department visit in last 30 days of life	More than 2 visits with location code or CPT code indicating emergency department use within 30 days before death	National Quality Forum[4]
Routine monitoring of digoxin in patients with congestive heart failure	Any measure of digoxin with no hospitalizations or emergency room visits during that year	National Health Service, UK[5]
Electroencephalogram (EEG) monitoring in individuals presenting with syncope	EEG on the same claim as diagnosis of syncope or at any time during the hospitalization with a code for syncope	National Health Service, UK[5]
Serological tests for helicobacter pylori	Any code indicating testing for H. pylori	National Health Service, UK[5]
Magnetic Resonance Imaging (MRI) in individuals with traumatic brain injury	MRI on the same claim as diagnosis if outpatient or during hospitalization if inpatient	National Quality Forum[4]

Indicator	Procedure	Reference
Positron emission tomography (PET), Computed Tomography (CT), and radionuclide bone scans in individuals with prostate cancer	PET, CT, or radionuclide bone scan AFTER diagnosis	Choosing Wisely[6]
Traction for low back pain	Traction with diagnosis of low back pain	Institute of Medicine[7]
Screening for asymptomatic carotid artery stenosis (CAS) in the general adult population	CPT code in outpatient setting	US Preventative Services Task Force[8]
Preoperative chest radiography in the absence of a clinical suspicion for intrathoracic pathology	CPT code in a 30-day window before an anesthesia code	Qaseem et al[9]
Performing tumor marker studies in asymptomatic women with previously treated breast cancer	CPT code with ICD code for breast cancer	Qaseem et al[9]
Don’t perform unproven diagnostic tests, such as immunoglobulin G (IgG) testing or an indiscriminate battery of immunoglobulin E (IgE) tests, in the evaluation of allergy	Use of CPT code on the same claim as a code for diagnoses in the denominator column	Choosing Wisely[6]
Don’t order sinus CT or indiscriminately prescribe antibiotics for uncomplicated acute rhinosinusitis	Any occurrence of sinus CT in the 3 months preceding the diagnosis of acute sinusitis	Choosing Wisely[6]
MRI Lumbar Spine for Low Back Pain	MRI of the lumbar spine studies with a diagnosis of low back pain without the patient having claims-based evidence of prior antecedent conservative therapy	Quality Net[10]
Thorax CT Use of Contrast Material	Number of thorax CT studies with and without contrast (“combined studies”)	Quality Net[10]
Abdomen CT use of contrast material	The number of Abdomen CT studies with and without contrast (“combined studies”)	Quality Net[10]

Reprinted by permission from Springer Nature: Springer, Journal of General Internal Medicine, Regional Supply of Medical Resources and Systemic Overuse of Health Care Among Medicare Beneficiaries, Mo Zhou, Allison H. Oakes, John F.P. Bridges et al, © 2018.

Table 1 References

1. Douglas PS, Garcia MJ, Haines DE, et al. ACCF/ASE/AHA/ASNC/HFSA/HRS/SCAI/SCCM/SCCT/SCMR 2011 Appropriate Use Criteria for Echocardiography. A Report of the American College of Cardiology Foundation Appropriate Use Criteria Task Force, American Society of Echocardiography, American Heart Association, American Society of Nuclear Cardiology, Heart Failure Society of America, Heart Rhythm Society, Society for Cardiovascular Angiography and Interventions, Society of Critical Care Medicine, Society of Cardiovascular Computed Tomography, Society for Cardiovascular Magnetic Resonance American College of Chest Physicians. J Am Soc Echocardiogr. 2011;24: 229–267.
2. Agency for Healthcare Research and Quality. National Guideline Clearinghouse. Available at: http://www.guideline.gov/index.aspx. Accessed July 9, 2018.
3. Blue Cross Blue Shield of Massachusetts. The Alternative QUALITY Contract. Available at: http://www.bluecrossma.com/visitor/pdf/alternative-quality-contract.pdf. Accessed July 9, 2018.
4. National Quality Forum. Quality Positioning System. Available at: https://www.qualityforum.org/QPS/. Accessed July 9, 2018.
5. National Institute for Health and Care Excellence (NICE). Do not do. Available at: https://www.nice.org.uk/about/what-we-do/into-practice. Accessed July 9, 2018.
6. ABIM Foundation. Choosing Wisely. 2018. Available at: http://www.choosingwisely.org/clinician-lists/. Accessed July 9, 2018.
7. Institute of Medicine. Knowing What Works in Healthcare: A Roadmap for the Nation. Washington, DC: The National Academies Press; 2008.
8. US Preventative Services Taskforce. Screening for carotid artery stenosis. 2007. Available at: http://www.uspreventiveservicestaskforce.org/uspstf/uspsacas.htm. Accessed July 9, 2018.
9. Qaseem A, Alguire P, Dallas P, et al. Appropriate use of screening and diagnostic tests to foster high-value, cost-conscious care. Ann Intern Med. 2012;156:147–149.
10. QualityNet. Imaging efficiency measures. Available at: https://www.qualitynet.org/dcs/ContentServer?cid=1228772297509&pagename=QnetPublic%2FPage%2FQnetTier3&c=Page. Accessed July 9, 2018.

Figure 1. Correlation Between the Unadjusted and Fully Adjusted Johns Hopkins Overuse Index

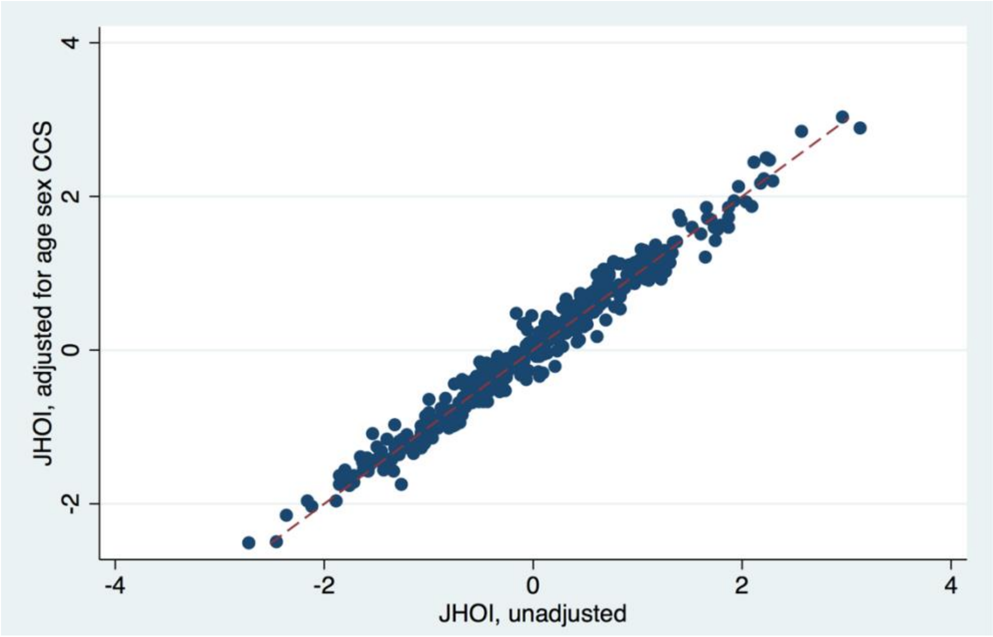

Association between the unadjusted Johns Hopkins Overuse Index and the age-, sex-, Clinical Conditions Software-adjusted Johns Hopkins Overuse Index (ρ=0.98, p<0.001). The dotted red line represents a perfect correlation.

Figure 2. Distribution of the Johns Hopkins Overuse Index in the first half of 2010

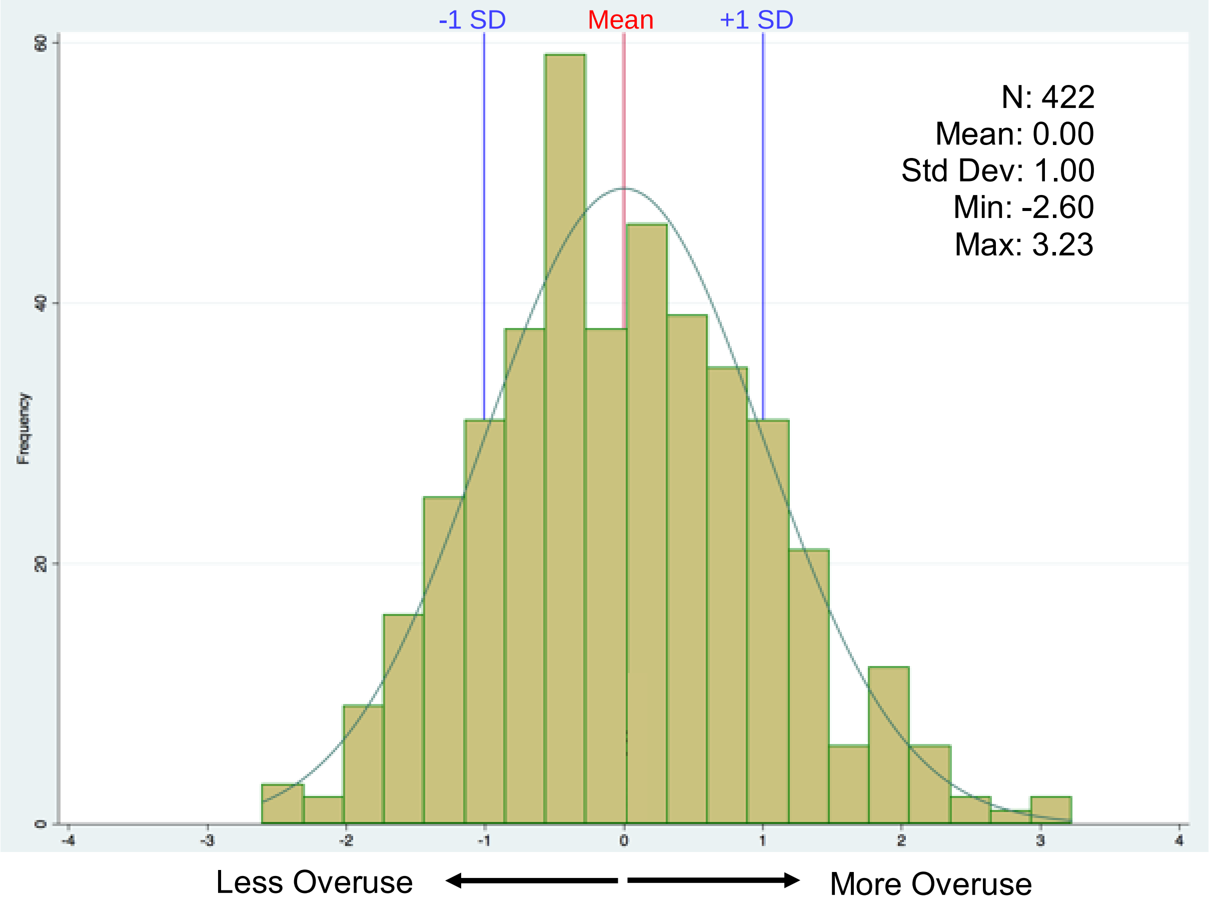

Figure 3-4. Correlation Between the Johns Hopkins Overuse Index and Meaningful Outcomes

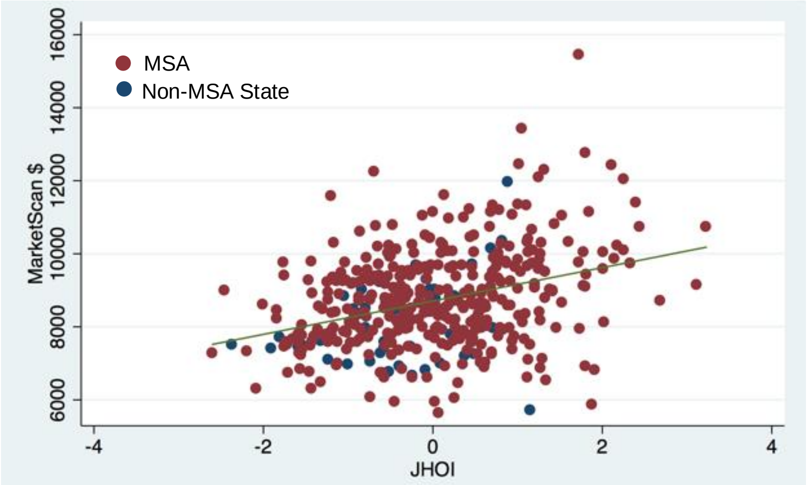

Association between the Johns Hopkins Overuse Index and Total Medicare per Capita Annual Costs in 2010 (ρ=0.32, p<0.001).

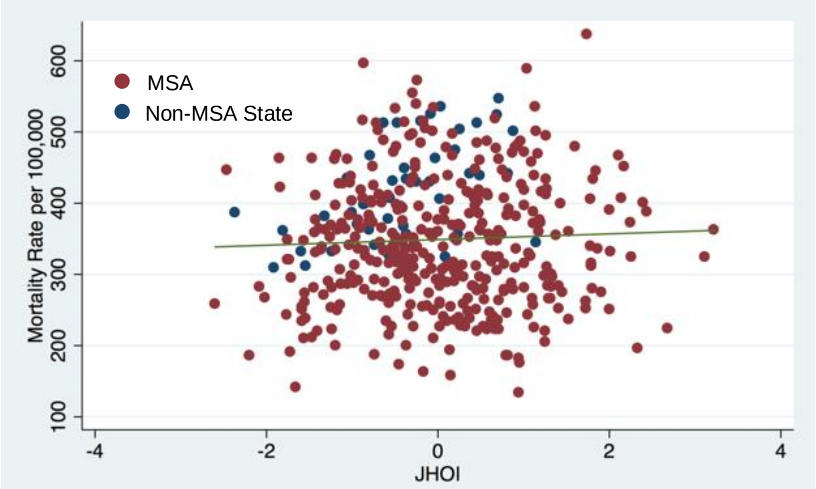

Lack of association between the Johns Hopkins Overuse Index and Mortality per 100,000 18-64 year old adults in 2010 (ρ=0.02, p=0.61).

Table 2. Stability over time

	2011a	2011b	2012a	2012b	2013a	2013b	2014a	2014b	2015a
2011a	1								
2011b	0.96	1							
2012a	0.95	0.95	1						
2012b	0.93	0.94	0.96	1					
2013a	0.90	0.89	0.93	0.93	1				
2013b	0.88	0.90	0.91	0.92	0.95	1			
2014a	0.86	0.87	0.88	0.89	0.90	0.93	1		
2014b	0.87	0.87	0.89	0.90	0.90	0.92	0.96	1	
2015a	0.79	0.80	0.82	0.82	0.85	0.85	0.90	0.91	1

Pairwise Spearman rank correlation results of semiannual JHOI estimates for January 2011 to June 2015. All models adjusted for age, sex.

Table 3. Persistent Regions

Persistently High Overusing Regions (N=37)		Persistently Low Overusing Regions (N=43)	
Bakersfield	CA	Rural	WI
Los Angeles-Long Beach-Glendale	CA	Eau Claire	WI
Madera	CA	Green Bay	WI
Oxnard-Thousand Oaks-Ventura	CA	Madison	WI
Redding	CA	Sheboygan	WI
San Jose-Sunnyvale-Santa Clara	CA	Wausau	WI
San Lois Obispo-Paso Robles-Arroyo Grande	CA	La Crosse-Onalaska	WI-MN
Santa Ana-Anaheim-Irvine	CA	Rural	NY
Santa Barbara-Santa Maria-Goleta	CA	Buffalo-Cheektowaga-Niagara Falls	NY
Visalia-Porterville	CA	Elmira	NY
Fort Lauderdale-Pompano Beach-Deerfield Beach	FL	Rochester	NY
Miami-Miami Beach-Kendall	FL	Syracuse	NY
Port St. Lucie	FL	Rural	MN
West Palm Beach-Boca Raton-Delray Beach	FL	Mankato-North Mankato	MN
Lafayette	LA	St. Cloud	MN
Lake Charles	LA	Duluth	MN-WI
Monroe	LA	Rural	IA
Brownsville-Harlingen	TX	Cedar Rapids	IA
Corpus Christi	TX	Waterloo-Cedar Falls	IA
Dallas-Plano-Irving	TX	Joplin	MO
Brunswick	GA	Springfield	MO
Savannah	GA	St. Joseph	MO-KS
Nassau County-Suffolk County	NY	Rural	ND
New York-Wayne-White Plains	NY-NJ	Bismarck	ND
Bend-Redmond	OR	Fargo	ND-MN
Medford	OR	Lewiston	ID-WA
Pittsburgh	PA	Oshkosh-Neenah	ID-WA
Scranton-Wilkes-Barre-Hazleton	PA	Rural	IL
Anchorage	AK	Danville	IL
Bridgeport-Stamford-Norwalk	CT	Kokomo	IN
Chicago-Naperville-Arlington Heights	IL	Muncie	IN
Las Vegas-Henderson-Paradise	NV	Rural	SD
Tulsa	OK	Sioux Falls	SD
		Fayetteville	NC
		Dothan	AL
		Grand Junction	CO
		Shreveport-Bossier City	LA
		Rural	ME
		Billings	MT
		Lima	OH
		Williamsport	PA
		Rural	VT

Persistently high or low overusing regions are defined as having a JHOI in the top or bottom quintile from 2011 to June 2015, respectively. Regions are listed by state frequency.
